# Habitat or temporal isolation: Unraveling herbivore–parasitoid speciation patterns using double digest RADseq

**DOI:** 10.1002/ece3.4457

**Published:** 2018-09-12

**Authors:** Y. Miles Zhang, Amber I. H. Bass, D. Catalina Fernández, Barbara J. Sharanowski

**Affiliations:** ^1^ Department of Biology University of Central Florida Orlando Florida; ^2^ Department of Biological Science University of Windsor Windsor Ontario Canada

**Keywords:** Braconidae, ddRADseq, herbivore, parasitic wasp, reproductive isolation

## Abstract

Ecological speciation is often observed in phytophagous insects and their parasitoids due to divergent selection caused by host‐associated or temporal differences. Most previous studies have utilized limited genetic markers or distantly related species to look for reproductive barriers of speciation. In our study, we focus on closely related species of *Lygus* bugs and two sister species of *Peristenus* parasitoid wasps. Using mitochondrial DNA *COI* and genomewide SNPs generated using ddRADseq, we tested for potential effects of host‐associated differentiation (HAD) or temporal isolation in this system. While three species of *Lygus* are clearly delineated with both *COI* and SNPs, no evidence of HAD or temporal differentiation was detected. Two *Peristenus* sister species were supported by both sets of markers and separated temporally, with *P. mellipes* emerging early in June and attacking the first generation of *Lygus*, while *P. howardi* emerging later in August and attacking the second generation of their hosts. This is one of the few studies to examine closely related hosts and parasitoids to examine drivers of diversification. Given the results of this study, the *Lygus*‐*Peristenus* system demonstrates temporal isolation as a potential barrier to reproductive isolation for parasitoids, which could indicate higher parasitoid diversity in regions of multivoltine hosts. This study also demonstrates that incorporating systematics improves studies of parasitoid speciation, particularly by obtaining accurate host records through rearing, carefully delimiting cryptic species and examining population‐level differences with genomic‐scale data among closely related taxa.

## 1 INTRODUCTION

A growing number of evolutionary studies have focused on ecological speciation, in which new species arise as a result of ecologically driven divergent selection (Egan et al., 2015; Hood et al., 2015; Nosil, Crespi, & Sandoval, 2002; Rundle & Nosil, 2005; Schluter, 2009). During ecological speciation, reproductive barriers arise as a by‐product of adaptation to divergent environments. Ecological speciation has been observed in herbivorous insects in the form of host‐associated differentiation (HAD), where specialists diverge through phenological or host shifts as a result of competition and/or predation (Nosil et al., 2002; Rundle & Nosil, 2005; Schluter, 2009). Adaptation to divergent host plants leads to an accumulation of multiple reproductive barriers, ultimately resulting in the separation and formation of new species (Dres & Mallet, 2002; Forbes et al., 2017). The presence of HAD is often associated with a temporal component, where temporal divergence in the breeding time over timescales ranging from days, seasons, or even years (Taylor & Friesen, 2017). Temporal isolation can contribute to divergence alone or concurrently with traits such as host preference to reinforce divergence along the speciation continuum (Egan et al., 2015; Feder et al., 1994; Taylor & Friesen, 2017). Although allopatric populations are often defined by spatial differentiation, populations with overlapping distributions and phenological differences can also be argued as allopatric, but on a temporal scale (Taylor & Friesen, 2017). Most documented cases of temporal speciation among phytophagous insects involve seasonal separation of breeding time after host shifts resulting in selection of synchrony with host phenology, contributing to reproductive isolation as selection (Egan et al., 2015; Feder et al., 1994; Nosil et al., 2002; Stireman, Nason, & Heard, 2005). These phenological shifts are often associated with genes controlling diapause duration, timing of diapause termination, and circadian rhythms, which could contribute to divergent selection that ultimately drives ecological speciation (Ragland, Egan, Feder, Berlocher, & Hahn, 2011; Ragland, Sim, Goudarzi, Feder, & Hahn, 2012; Ragland et al., 2017; Taylor & Friesen, 2017).

Numerous studies have shown that generalist insect herbivore “species” are often multiple, genetically divergent cryptic lineages, each specializing on a subset of the full host plant range (Dres & Mallet, 2002; Peccoud, Ollivier, Plantegenest, & Simon, 2009; Powell, Forbes, Hood, & Feder, 2014). This is an important distinction as true generalists feed on a variety of host plants indiscriminately, while cryptic specialists exhibit host preferences that were overlooked due to morphological similarities. Therefore, the accurate identification of true generalists from cryptic specialists in various stages of speciation is vital to studies on the effects of host or temporal differences on biogenesis.

HAD has been recorded in diverse insect families across multiple orders (Antwi, Sword, & Medina, 2015; Ferrari, West, Via, & Godfray, 2012; Leppanen, Malm, Varri, & Nyman, 2014; Sword, Joern, & Senior, 2005), further suggesting that it is an important driver of speciation that contributed to the insect biodiversity we see today. In addition, HAD can have rippling effects at higher trophic levels, resulting in divergence of parasitoids in the form of cascading/sequential HAD (Abrahamson & Weis, 1997; Forbes, Powell, Stelinski, Smith, & Feder, 2009; Hood et al., 2015; Nicholls, Schönrogge, Preuss, & Stone, 2018; Stireman, Nason, Heard, & Seehawer, 2006). As many parasitoids are also cryptic specialists that are tightly linked to the phenology of their hosts, cascading HAD on species lineages of herbivores could result in the sequential radiation of these hyperdiverse lineages of parasitoids (Forbes et al., 2009; Hood et al., 2015; Stireman et al., 2006). However, many previous studies of HAD and sequential HAD were limited to few molecular markers (Antwi et al., 2015; Hood et al., 2015; Leppanen et al., 2014; Nicholls et al., 2018; Stireman et al., 2006), which provides limited molecular characters to examine fine‐scaled species‐level differentiation. In addition, most studies focus on specialist herbivores with few studies on parasitoids. Studies that have involved examinations of parasitoids have mainly included assemblages of distantly related parasitoids that make inferences about drivers of diversification in upper trophic levels difficult (Hood et al., 2015; Nicholls et al., 2018; Stireman et al., 2006). Therefore, studies focusing on closely related parasitoids species are needed to examine patterns of speciation due to ecologically divergent selection.

Accurate delimitation of divergent lineages is paramount to speciation studies, as they are often morphologically cryptic. Studies utilizing variations in restriction‐site associated DNA sequencing (RADseq) to delimit species and determine drivers of divergence have become more abundant (Bagley, Sousa, Niemiller, & Linnen, 2017; Bernal, Gaither, Simison, & Rocha, 2017; de Oca et al., 2017; Eaton & Ree, 2013). RADseq approaches are less susceptible to incomplete lineage sorting and introgression than traditional multigene methods (Andrews, Good, Miller, Luikart, & Hohenlohe, 2016). This method is ideal for detecting population/species‐level differences and has been shown to be promising for studies on ecological speciation of herbivorous insects (Bagley et al., 2017; Egan et al., 2015).

Studying the reproductive barriers of parasitoid in relation to their hosts is central to understanding origins of parasitoid diversity and may also provide important insights into conservation biology as parasitoids have been shown to provide ecosystems with trophic redundancy that reduces extinction risks (Sanders, Thébault, Kehoe, & van Veen, 2018). In addition, understanding the intimate relationships between pestiferous herbivores and their parasitoids would greatly improve the success rate of biological control programs (Peixoto et al., 2018; Zhang, Ridenbaugh, & Sharanowski, 2017). To that end, we investigate potential reproductive barriers in the *Lygus*‐*Peristenus* system, which includes a genus of economically important herbivores and the parasitoid species that attack them.

The herbivores in this system are plant bugs in the genus *Lygus* Hahn (Hemiptera: Miridae), which include many species of generalist agricultural pests (such as *Lygus lineolaris* Palisot de Beauvois) that feed on a variety of economically important crops. Although HAD has been recorded from other Miridae (Hereward, Walter, Debarro, Lowe, & Riginos, 2013), no evidence of HAD has been shown in *Lygus* species despite the detection of population‐level differences based on geography (Burange, Roehrdanz, & Boetel, 2012; Zhou, Kandemir, Walsh, Zalom, & Lavine, 2012). *Lygus* have one to three generations per year depending on the temperature, where southern populations in warmer climates are multivoltine and northern populations in cooler climates tend to be univoltine (Cárcamo et al., 2002; Haye et al., 2013). The Canadian prairies ecosystem is a major agricultural growing region where *Lygus* is an economically relevant pest on several field crops, such as canola, alfalfa, and mustard. Closely related species are often found in sympatry, so HAD may be a driver of population divergence in this system, as populations could be cryptic, specializing on certain plants.

Species of *Peristenus* (Hymenoptera: Braconidae) are widely distributed koinobiont endoparasitoids of nymphal plant bugs, including *Lygus* species (Zhang, Stigenberg, Meyer, & Sharanowski, 2018). A recent revision of the Nearctic *Peristenus pallipes* complex synonymized nine species recognized by Goulet and Mason (2006) to just three based on morphometrics, mitochondrial DNA (*COI* and *CytB*), and ecological differences (Zhang et al., 2017). This revision also demonstrated a range overlap for *Peristenus dayi* Goulet with sister species *Peristenus mellipes* (Cresson) and *Peristenus howardi* Shaw in southern Alberta (Zhang et al., 2017). As these *Peristenus* species persist in sympatry, there are likely reproductive barriers preventing hybridization and interbreeding between species. These may be ecological isolating mechanisms, such as differences in micro‐habitat, emergence timing, and reproduction. *Peristenus* host preference may also explain the maintenance of three sympatric species, but due to morphological similarity among *Lygus* nymphs, host records are often listed simply as *Lygus* species (Goulet & Mason, 2006). The drivers and maintenance of species boundaries in these closely related parasitoids are unknown, but a likely explanation is divergence through sequential HAD as their hosts specialize and diverge.

In this study, we used a combination of *COI* (mtDNA) and double digest RADseq (ddRADseq) (Peterson, Weber, Kay, Fisher, & Hoekstra, 2012) to test for barriers of reproductive isolation in closely related parasitoids. We (a) confirm monophyly and delimit species of *Lygus* and their *Peristenus* parasitoids; (b) test for potential host plant associations or temporal differentiation on sympatric species of *Lygus*; and (c) determine whether sequential HAD or temporal differentiation are driving forces of speciation on sympatric species of *Peristenus*. As herbivore–parasitoid evolutionary histories can provide valuable insights into the genesis of biodiversity, this is one of the first studies to address the evolutionary patterns within a tritrophic system that utilizes host plant, herbivore, and parasitoid using next‐generation sequencing data and closely related parasitoids.

## 2 MATERIALS AND METHODS

### 2.1 Sample collection and DNA Extraction

To obtain *Peristenus* with accurate host records delineated to species, we sampled early instar nymphal *Lygus* bugs weekly from May to August of 2015 from two sites in Lethbridge, Alberta, as this is the only region in which the range of both *P. mellipes* and *P. howardi* overlaps (Sharanowski, Zhang, & Wanigasekara, 2014; Zhang et al., 2017). One additional site where only *P. mellipes* is found was sampled in Carman, Manitoba. While *Lygus* attacks a variety of plants, we chose three common host plants: alfalfa (*Medicago sativa* L.), yellow sweetclover [*Melilotus officinalis* (L.)], and wild mustard (*Sinapis arvensis* L.) as they were readily accessible and yielded large quantities of nymphs based on pilot studies. We reared nymphs individually in growth chambers (25°C, 14:10 hr L:D photoperiod) using green beans as a food source and checked daily for parasitoid emergence. If the *Lygus* nymphs were parasitized, the emerged larval parasitoid and dead host were preserved in 95% EtOH until DNA extraction. Genomic DNA was extracted following the DNeasy Tissue Kit Protocol (Qiagen, Valencia, CA, USA), using a destructive sampling method as the larval parasitoid and host nymphs were unidentifiable morphologically. We quantified the concentration of DNA extracts using Quant‐iT High‐Sensitivity DNA Assay Kit (Invitrogen, Eugene, OR, USA). *Peristenus dayi* was excluded from this study despite being closely related to the other parasitoids, as it parasitizes *Adelphocoris lineolatus* (Goeze), a distant relative of *Lygus* within Miridae, and we were interested in patterns between closely related herbivores and parasitoids.

### 2.2 Molecular data protocols

We amplified the mitochondrial gene cytochrome oxidase I (*COI*) using universal primers LCO1490 (5′‐GGT CAA CAA ATC ATA AAG ATA TTG G‐3′) and HCO2198 (5′‐TAA ACT TCA GGG TGA CCA AAA AAT CA‐3′) (Folmer, Black, Hoeh, Lutz, & Vrijenhoek, 1994). Polymerase chain reactions were performed on a Bio‐Rad MyCycler thermal cycler (Hercules, CA, USA), using ~1 μg DNA extract, 1× Standard Taq Buffer (10 mM Tris–HCl, 50 mM KCl, 1.5 mM MgCl_2_, pH 8.3; New England Biolabs, Ipswich, Massachusetts, USA), 200 μM dNTP (Invitrogen, Carlsbad, California, USA), 4 mM MgSO_4_, 400 nM of each primer, 1 unit of Taq DNA polymerase (New England Biolabs), and nuclease‐free water to a final volume of 25 μl.

We generated *COI* amplicons for both *Lygus* and *Peristenus* with an initial denaturation of 1min at 95°C, followed by 35 cycles of 95°C for 15 s, 49°C for 15 s, and 72°C for 45 s, and a final elongation period of 4 min at 72°C. Reaction products were cleaned with Agencourt CleanSEQ magnetic beads (Beckman Coulter Life Sciences, Indianapolis, IN, USA) and sequenced in both directions using the BigDye Terminator Cycle Sequencing Kit (Applied Biosystems, Foster City, CA, USA) and the Applied Biosystems 3730xl DNA Analyzer at the University of Kentucky, Advanced Genetic Technologies Center (UK‐AGTC). Contigs were assembled and edited using Geneious version 8.18 (Kearse et al., 2012), and alignment was conducted using MUSCLE under default settings (Edgar, 2004) and checked manually by eye using the reading frame as a guide. All *COI* sequences were uploaded to GenBank (accession nos. MG944319–MG944389).

We used a modified ddRADseq protocol from Peterson et al. (2012) to generate genomewide SNPs for both *Lygus* and *Peristenus*. NlaIII and MluCl (NEB, Ipswich, MA, USA) were the enzyme pair chosen based on in silico digestion of the following genomes: *Acyrthosiphon pisum* (International Aphid Genomics Consortium, 2010), *Microplitis demolitor* (Burke, Walden, Whitfield, Robertson, & Strand, 2014), and *Fopius arisanus* (Geib, Liang, Murphy, & Sim, 2017) using SimRAD (Lepais & Weir, 2014). We prepared libraries containing up to 48 individuals grouped by DNA yield, with each sample assigned one of 48 unique 5‐base pair (bp) in‐line barcode sequences during adapter ligation. Each set of 48 samples was then pooled for automated size selection (216–336 bp fragments) on a PippinHT (Sage Science, Beverly, MA, USA). The size‐selected samples were then subjected to 12 rounds of high‐fidelity PCR amplification (Q5 High‐Fidelity DNA Polymerase, NEB) using PCR primers that included one of 12 unique Illumina multiplex read indices. After verifying library quality using high‐sensitivity DNA kit on TapeStation (Agilent, Santa Clara, CA, USA), libraries were sent to Sanford Burnham Prebys Medical Discovery Institute (Orlando, FL, USA) for sequencing using 2 × 300 bp paired‐end reads on a single Illumina MiSeq lane. All raw fastq files were uploaded onto the NCBI SRA database accession number SRP132595.

We used ipyrad v0.7.23 (Eaton, 2014) to process raw sequences, using the following stringent settings to ensure the data quality for downstream analyses after parsing out *Lygus* from *Peristenus*: Assembly methods: de novo; minimum depth of reads per within‐sample cluster: 10; maximum number of sites in a read which can have a quality score of less than twenty: 4; clustering threshold: 0.90; minimum number of samples in each across‐sample cluster: 10; maximum number of individuals with a shared heterozygous site in an across‐sample cluster: 3. These settings were chosen based on multiple test runs with different parameter settings to balance between stringent filtering high‐quality SNP calls without losing too much data. All other settings were default values. Additionally, we removed samples with >80% missing data and suspected *Peristenus* males, which are haploid and thus have low heterozygosity.

### 2.3 Phylogenetic analyses

The best‐fitting model of molecular evolution for *COI* was tested using jmodeltest2 (Darriba, Taboada, Doallo, & Posada, 2012). The general time‐reversible model, with a parameter for invariant sites and rate heterogeneity modeled under a gamma distribution (GTR + I + Γ), was chosen based on the Bayesian information criterion (BIC). The *COI* sequences were then analyzed using MrBayes v 3.2.6 (Ronquist et al., 2012) on the CIPRES Science Gateway (Miller et al., 2010). Two independent searches were carried out and four chains run for 2,000,000 generations, sampling every 1,000th generation and with a 10% burn‐in discarded. The dataset was not partitioned based on the nucleotide position as it would limit the amount of data needed for accurate parameter estimation. The phylogenetic trees were visualized in FigTree v1.4.2 (Rambaut, 2012) and modified using R package ggtree (Yu, Smith, Zhu, Guan, & Lam, 2017). The *Lygus* samples were identified by comparing *COI* sequences with identified adult specimens on the Barcode of Life database (BOLD: http://barcodinglife.org/) that were authoritatively identified by *Lygus* expert Michael D. Schwartz. In cases of ambiguous identification (i.e., multiple species share the same DNA barcode), we chose the species name based on the most common identification (>80%) for each species. Similarly, *Peristenus* was identified by comparing the *COI* sequences with samples from Zhang et al. (2017).

A maximum‐likelihood supermatrix approach using the concatenated ddRADseq SNPs dataset was also conducted with RAxML 8.2.0 (Stamatakis, 2006), using the GTR + Γ model of nucleotide substitution and 1,000 bootstrap pseudoreplicates. The resulting trees were visualized and modified in the same manner as the *COI* trees.

### 2.4 Population genomic analyses

To determine whether there was population structure within clades identified in the phylogenetic analysis, we performed a Bayesian clustering analysis for both *Lygus* and *Peristenus* unlinked SNP datasets (1 SNP per locus) from the ipyrad output stated earlier without prior assignments in Structure v 2.3.4 (Pritchard, Stephens, & Donnelly, 2000). Ten runs were completed for each population (*K*) up to the maximum number of populations within each clade using 100,000 burn‐ins and 500,000 replicates for each run. The R package pophelper (Francis, 2017) was used to visualize the diagrams. The Evanno Δ*K* method (Evanno, Regnaut, & Goudet, 2005) was used in Structure Harvester v 0.6.94 (Earl, 2012) to determine the most likely value for *K*. We also created a custom dataset of the SNPs containing only Alberta populations of *P. mellipes* and *P. howardi* in ipyrad using the same settings discussed above. We tested for potential genetic differences under selection between the Alberta populations where the two *Peristenus* species are found in sympatry.

Impacts of locality, host association, and time of emergence on genetic variation of the three *Lygus* species were tested using AMOVA (analysis of molecular variance) using clustering between localities (for *L. borealis*), host plants (for *L. keltoni* and *L. elisus*), and collecting dates for all three species of *Lygus*. Similarly, AMOVA was used to test for differences between hosts for both *Peristenus* species and difference between collection localities for *P. mellipes*. All AMOVAs were conducted with R packages adegenet (Jombart & Ahmed, 2011) and poppr (Kamvar, Tabima, & Grünwald, 2014) using the full SNP dataset as described above.

## 3 RESULTS

### 3.1 Phylogenetic analyses

A total of 23 samples each of *Lygus* and *Peristenus* were used to generate the ddRADSeq dataset (Table 1). There were an average of ~732,000 reads per individual with a mean length of 142 bp and 15× mean depth of coverage per loci (average 183 loci per *Lygus* and 5,993 loci per *Peristenus*). The final filtered SNP dataset consisted of 14 of 23 *Lygus* individuals with 1,453 parsimonious informative SNPs and 19 of 23 *Peristenus* individuals with 18,157 parsimonious informative SNPs (Table 1). The low number of SNPs recovered from *Lygus* was likely due to the low‐input DNA quantity or degradation because of parasitism by *Peristenus*. The topology of the maximum‐likelihood trees based on the ddRADseq data recovered the same clades as the *COI* Bayesian analyses with strong bootstrap support for all three species of *Lygus* (Supporting information Figure S1) and both species of *Peristenus* (Supporting information Figure S2).

**Table 1:** Sampling information for *Lygus* nymphs and the *Peristenus* that emerged from the nymphs

*Lygus* sample number	ID	GenBank/SRA Accession number	*Peristenus* Sample number	ID	GenBank/SRA Accession number	Locality	Host plant	Date collected
YMZ213	*L. borealis*	MG944319/SAMN08614153	N/A	N/A	N/A	Manitoba, Carman, 49.500834, −98.023839	Alfalfa	16.VI.2015
N/A	N/A	N/A	YMZ224	*P. mellipes*	MG944353	Manitoba, Carman, 49.500834, −98.023839	Alfalfa	16.VI.2015
YMZ215	*L. borealis*	MG944320	YMZ225	*P. mellipes*	MG944354	Manitoba, Carman, 49.500834, −98.023839	Alfalfa	16.VI.2015
YMZ216	*L. borealis*	MG944321	YMZ226	*P. mellipes*	MG944355/SAMN08614174	Manitoba, Carman, 49.500834, −98.023839	Alfalfa	16.VI.2015
YMZ217	*L. borealis*	MG944322	YMZ227	*P. mellipes*	MG944356	Manitoba, Carman, 49.500834, −98.023839	Alfalfa	16.VI.2015
YMZ218	*L. borealis*	MG944323/SAMN08614154	YMZ228	*P. mellipes*	MG944357/SAMN08614175	Manitoba, Carman, 49.500834, −98.023839	Alfalfa	16.VI.2015
YMZ220	*L. borealis*	MG944324/SAMN08614155	YMZ230	*P. mellipes*	MG944358/SAMN08614176	Manitoba, Carman, 49.500834, −98.023839	Alfalfa	16.VI.2015
YMZ221	*L. borealis*	MG944325	YMZ231	*P. mellipes*	MG944359/SAMN08614177	Manitoba, Carman, 49.500834, −98.023839	Alfalfa	16.VI.2015
YMZ222	*L. borealis*	MG944326	YMZ232	*P. mellipes*	MG944360/SAMN08614178	Manitoba, Carman, 49.500834, −98.023839	Alfalfa	16.VI.2015
YMZ233	*L. keltoni*	MG944327	YMZ243	*P. mellipes*	MG944361/SAMN08614179	Alberta, Lethbridge, 49.721307, −112.853001	Yellow Clover	30.VI.2015
YMZ234	*L. elisus*	MG944328/SAMN08614156	YMZ244	*P. mellipes*	MG944362	Alberta, Lethbridge, 49.721307, −112.853001	Yellow Clover	30.VI.2015
YMZ235	*L. keltoni*	MG944329	YMZ245	*P. mellipes*	MG944363/SAMN08614180	Alberta, Lethbridge, 49.721307, −112.853001	Yellow Clover	30.VI.2015
YMZ236	*L. borealis*	MG944330	YMZ246	*P. mellipes*	MG944364/SAMN08614181	Alberta, Lethbridge, 49.700244, −112.763226	Alfalfa	30.VI.2015
YMZ237	*L. borealis*	MG944331	YMZ247	*P. howardi*	MG944365/SAMN08614167	Alberta, Lethbridge, 49.700244, −112.763226	Alfalfa	08.VIII.2015
N/A	N/A	N/A	YMZ248	*P. howardi*	MG944366	Alberta, Lethbridge, 49.700244, −112.763226	Alfalfa	08.VIII.2015
YMZ239	*L. elisus*	MG944332/SAMN08614157	YMZ249	*P. mellipes*	MG944367/SAMN08614182	Alberta, Lethbridge, 49.700244, −112.763226	Alfalfa	30.VI.2015
N/A	*L. borealis*	N/A	YMZ250	*P. mellipes*	MG944368	Alberta, Lethbridge, 49.700244, −112.763226	Alfalfa	30.VI.2015
YMZ241	*L. borealis*	MG944333/SAMN08614158	YMZ251	*P. mellipes*	MG944369/SAMN08614183	Alberta, Lethbridge, 49.700244, −112.763226	Alfalfa	30.VI.2015
N/A	N/A	N/A	YMZ252	*P. mellipes*	MG944370	Alberta, Lethbridge, 49.700244, −112.763226	Alfalfa	30.VI.2015
N/A	N/A	N/A	YMZ263	*P. howardi*	MG944371	Alberta, Lethbridge, 49.721307, −112.853001	Wild Mustard	08.VIII.2015
N/A	N/A	N/A	YMZ264	*P. howardi*	MG944372	Alberta, Lethbridge, 49.721307, −112.853001	Wild Mustard	08.VIII.2015
YMZ255	*L. keltoni*	MG944334/SAMN08614159	YMZ265	*P. howardi*	MG944373/SAMN08614168	Alberta, Lethbridge, 49.721307, −112.853001	Wild Mustard	08.VIII.2015
YMZ256	*L. elisus*	MG944335	N/A	N/A	N/A	Alberta, Lethbridge, 49.721307, −112.853001	Wild Mustard	08.VIII.2015
YMZ257	*L. keltoni*	MG944336/SAMN08614160	YMZ267	*P. howardi*	MG944374/SAMN08614169	Alberta, Lethbridge, 49.721307, −112.853001	Wild Mustard	08.VIII.2015
YMZ259	*L. keltoni*	MG944337	YMZ269	*P. howardi*	MG944375	Alberta, Lethbridge, 49.721307, −112.853001	Wild Mustard	08.VIII.2015
YMZ260	*L. keltoni*	MG944338/SAMN08614161	YMZ270	*P. howardi*	MG944376/SAMN08614169	Alberta, Lethbridge, 49.721307, −112.853001	Alfalfa	08.VIII.2015
YMZ262	*L. elisus*	MG944339	YMZ271	*P. howardi*	MG944377/SAMN08614170	Alberta, Lethbridge, 49.721307, −112.853001	Alfalfa	08.VIII.2015
YMZ293	*L. borealis*	MG944340	YMZ303	*P. mellipes*	MG944378	Manitoba, Carman, 49.500834, −98.023839	Alfalfa	16.VI.2015
YMZ294	*L. borealis*	MG944341	YMZ304	*P. mellipes*	MG944379	Manitoba, Carman, 49.500834, −98.023839	Alfalfa	16.VI.2015
YMZ295	*L. borealis*	MG944342	N/A	N/A	N/A	Manitoba, Carman, 49.500834, −98.023839	Alfalfa	16.VI.2015
YMZ296	*L. borealis*	MG944343	N/A	N/A	N/A	Manitoba, Carman, 49.500834, −98.023839	Alfalfa	16.VI.2015
YMZ297	*L. borealis*	MG944344	YMZ307	*P. mellipes*	MG944380	Manitoba, Carman, 49.500834, −98.023839	Alfalfa	16.VI.2015
YMZ298	*L. borealis*	MG944345	YMZ308	*P. mellipes*	MG944381	Manitoba, Carman, 49.500834, −98.023839	Alfalfa	16.VI.2015
YMZ299	*L. borealis*	MG944346	N/A	N/A	N/A	Manitoba, Carman, 49.500834, −98.023839	Alfalfa	16.VI.2015
YMZ300	*L. borealis*	MG944347	N/A	N/A	N/A	Manitoba, Carman, 49.500834, −98.023839	Alfalfa	16.VI.2015
YMZ301	*L. borealis*	MG944348/SAMN08614161	YMZ311	*P. mellipes*	MG944382	Manitoba, Carman, 49.500834, −98.023839	Alfalfa	16.VI.2015
YMZ302	*L. borealis*	MG944349	YMZ322	*P. howardi*	MG944383/SAMN08614184	Manitoba, Carman, 49.500834, −98.023839	Alfalfa	08.VIII.2015
YMZ313	*L. elisus*	SAMN08614163	YMZ323	*P. howardi*	MG944384/SAMN08614172	Alberta, Lethbridge, 49.700244, −112.763226	Alfalfa	08.VIII.2015
YMZ314	*L. borealis*	MG944350/SAMN08614164	YMZ325	*P. howardi*	MG944385/SAMN08614173	Alberta, Lethbridge, 49.700244, −112.763226	Alfalfa	08.VIII.2015
YMZ316	*L. elisus*	SAMN08614165	N/A	N/A	N/A	Alberta, Lethbridge, 49.700244, −112.763226	Alfalfa	08.VIII.2015
YMZ317	*L. borealis*	MG944351	YMZ327	*P. howardi*	MG944386	Alberta, Lethbridge, 49.700244, −112.763226	Alfalfa	08.VIII.2015
N/A	N/A	N/A	YMZ329	*P. mellipes*	MG944387	Alberta, Lethbridge, 49.700244, −112.763226	Alfalfa	30.VI.2015
N/A	N/A	N/A	YMZ330	*P. mellipes*	MG944388	Alberta, Lethbridge, 49.700244, −112.763226	Alfalfa	30.VI.2015
YMZ331	*L. keltoni*	MG944352/SAMN08614166	YMZ332	*P. mellipes*	MG944389/SAMN08614185	Alberta, Lethbridge, 49.700244, −112.763226	Alfalfa	16.VI.2015

Note

GenBank Accession Numbers for *COI* and SRA Accession Numbers for ddRADseq are provided when available.

A total of 33 *Lygus* (543 bp) and 37 *Peristenus* (629 bp) *COI* sequences were used for the phylogenetic analyses (Table 1). Three monophyletic clades of *Lygus* were identified based on the monophyletic clustering with identified specimens available in BOLD: *Lygus borealis* (Kelton), *Lygus keltoni* Schwartz, and *Lygus elisus* Van Duzee (Supporting information Figure S1). All three species of *Lygus* were collected in Alberta, while only *L. borealis* was collected in Manitoba. Both *L. keltoni* and *L. elisus* were collected from all three host plants, while *L. borealis* was collected exclusively on alfalfa (Table 1). Both *Peristenus mellipes and P. howardi* were recovered as monophyletic clades (Supporting information Figure S2). *Peristenus mellipes* was reared from all three *Lygus* species and found in both Manitoba and Alberta, while *P. howardi* was reared from *L. borealis* and *L. keltoni* and was found exclusively in Alberta (Table 1; Supporting information Figure S2).

### 3.2 Population genomic analyses

Using the Δ*K* approach, Bayesian clustering analyses in STRUCTURE indicated *K* = 3 (Figure 3a) in *Lygus*, which corresponds to the number of species identified by phylogenetic methods (Figure 2). The STRUCTURE results show *K* = 3 among the two *Peristenus* species, as population structure was not found within *P. howardi*, but splits *P. mellipes* into an Alberta‐specific population and a Manitoba population (Figure 3B).

No significant genetic differentiation was detected among any of the AMOVA partitions (locality, host plant, collecting date) for the three *Lygus* species (Table 2). No differences between host bugs were detected for both species of *Peristenus* (Table 3a), but significant genetic differences (*p* = 0.01) were detected among collection localities within *P. mellipes*, explaining 11.77% of the genetic variation (Table 3b).

**Table 2:** Analysis of molecular variance (AMOVA) using clustering between (a) localities, (b) host plants, and (c) collecting dates for all three species of *Lygus* used in this study

Taxon assessed	Source of variation	*df*	Variance component	% total variation	Ф‐statistics	*p*‐value
(a) Between localities						
*L. borealis*	Between localities	1	−1.55	−5.95	−0.73	0.95
	Among samples within localities	4	−17.74	−67.79	−0.64	1.00
	Within samples	6	46.48	173.74	−0.06	1.00
(b) Between host plants						
*L. keltoni*	Among plants	1	1.09	3.01	−0.73	0.37
	Among samples within plants	2	−27.61	−76.09	−0.78	0.89
	Within samples	4	62.79	173.08	10.03	0.98
*L. elisus*	Among plants	1	0.20	0.64	‐0.87	0.71
	Among samples within plants	3	−27.90	−87.95	−0.89	0.93
	Within samples	4	59.43	187.31	0.01	1.00
(c) Between collection dates						
*L. borealis*	Among dates	1	−2.21	−8.69	−0.78	0.87
	Among samples within dates	4	−17.84	−70.15	−0.64	0.99
	Within samples	6	45.48	178.83	−0.09	1.00
*L. keltoni*	Among dates	1	1.16	3.17	−0.72	0.45
	Among samples within dates	2	−27.46	−75.24	−0.78	0.96
	Within samples	4	62.79	172.07	0.03	1.00
*L. elisus*	Among dates	1	−0.87	−2.78	−0.91	1.00
	Among samples within dates	2	−27.37	−87.74	−0.85	1.00
	Within samples	4	59.43	190.52	−0.03	1.00

**Table 3:** (a) Analysis of molecular variance (AMOVA) using clustering between different localities and different host bugs for all both species of *Peristenus* used in this study. (b) Hierarchical AMOVA of collection localities grouped within host bug and host bugs grouped within localities for *Peristenus mellipes*

Taxon assessed	Source of variation	*df*	Variance component	% total variation	*Ф*‐statistics	*p*‐value
(a) Between host bugs						
*P. mellipes*	Between bugs	2	110.19	12.42	0.44	0.11
	Among samples within bugs	8	282.52	31.84	0.36	**0.01**
	Within samples	11	494.54	55.74	0.12	**0.01**
*P. howardi*	Between bugs	2	−12.10	−2.44	0.37	0.66
	Among samples within bugs	5	193.81	39.03	0.38	**0.02**
	Within samples	8	314.86	63.41	−0.02	**0.01**
(b) Between localities						
*P. mellipes*	Between localities	1	104.39	11.77	0.44	**0.01**
	Among samples within localities	9	287.69	32.45	0.37	**0.02**
	Within samples	11	494.54	55.78	0.12	**0.01**

*Note*

Significant *p*‐values are bolded.

## 4 DISCUSSION

### 4.1 Identification of *Lygus* and *Peristenus* species using molecular data

The accurate identification of *Lygus* species has been problematic in the past, because of the inconsistency between morphological differences of nymphs and *COI* data (Gwiazdowski, Foottit, Maw, & Hebert, 2015). The *Lygus* species included in this study, *L. borealis*,* L. elisus*, and *L. keltoni*, were often misidentified even by experts because of their variable adult phenotypes (Gwiazdowski et al., 2015). This taxonomic confusion has made previous host plant records in this group unreliable. Using *COI* and SNPs, we confirmed the identity of the *Lygus* nymphs used in this study and established accurate host bug records for the parasitoids. Taxonomic revision of *Lygus* is needed, as current morphological character without the aid of molecular tools is unreliable, and we advise caution when using publicly available databases such as GenBank and BOLD as misidentifications are common despite expert identification. The identification of *P. mellipes* and *P. howardi* using both *COI* and SNPs was consistent with Zhang et al. (2017), lending support to the continued use of *COI* to accurately delimit closely related parasitoid wasps at a cheaper cost compared to genomic data.

### 4.2 Lack of HAD and Temporal isolation within *Lygus* species

**Figure 1:**
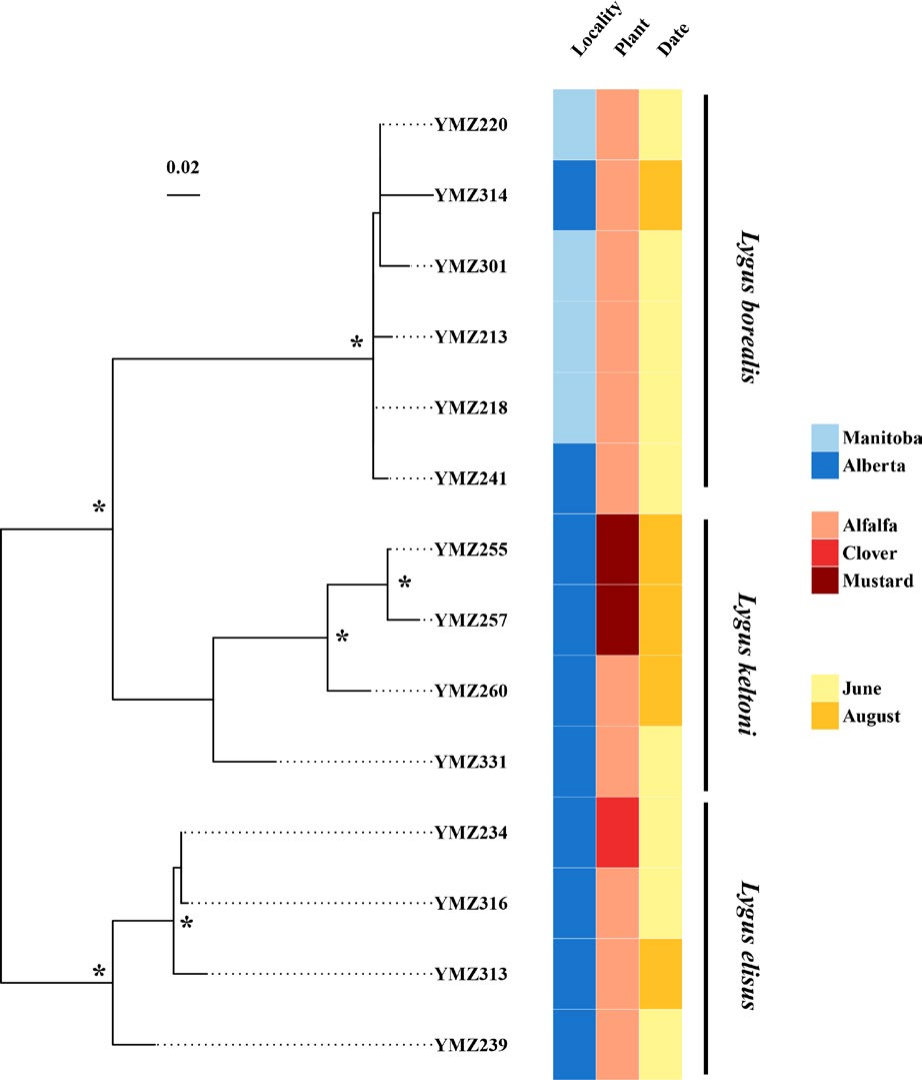
Inferred phylogeny of *Lygus* species from the RAxML analysis of the SNP data. Asterisk indicates bootstrap value of ≥90. Sampling locality is color‐coded in shades of blue, host plant in shades of red, and collecting date in shades of yellow

Based on our phylogenetic analyses on *Lygus* (Figure 1) and AMOVA (Table 2), it is unlikely that *Lygus* species evolved through host‐associated differentiation in the Canadian prairies. The three species of *Lygus* are all generalist herbivores feeding on a variety of available food sources, as no host plant‐specific lineages were found within each species (Figure 1, Table 2). While both *L. elisus* and *L. keltoni* were found on all three host plants sampled in this study, *L. borealis* were only found from alfalfa. The apparently narrow host range of *L. borealis* could be a by‐product of our sampling, as they have been collected from other host plants such as canola (*Brassica* spp.) in other studies (Cárcamo et al., 2002; Otani & Cárcamo, 2011). These results show that *Lygus* species are truly generalists as we found no genetic divergence based on host. This lack of HAD is consistent with studies of other *Lygus* species such as *L. lineolaris* (Burange et al., 2012) and *L. hesperus* (Zhou et al., 2012) despite the detection of population‐level differences, indicating that factors other than HAD likely drove their evolution.

### 4.3 Temporal isolation but no HAD within the *Peristenus* species

**Figure 2:**
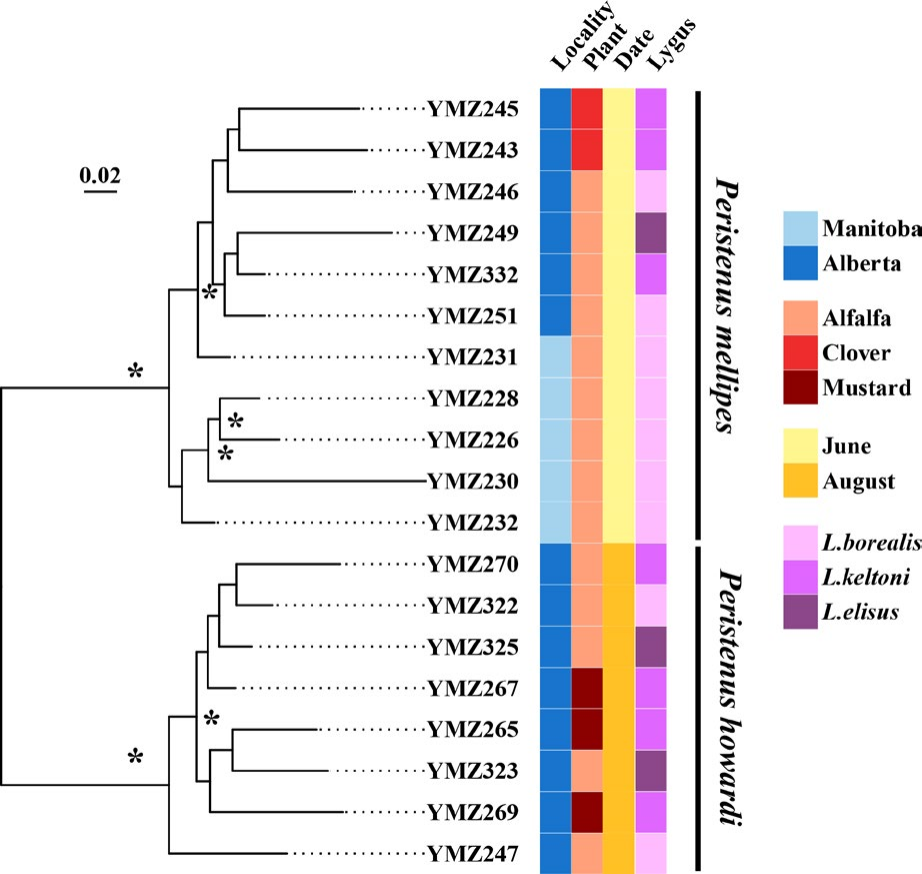
Inferred phylogeny of *Peristenus* species from the RAxML analysis of the SNP data. Asterisk indicates bootstrap value of ≥90. Sampling locality is color‐coded in shades of blue, host plant in shades of red, collecting date in shades of yellow, and host bug in shades of purple

*Peristenus* host choice was not significantly different in the hierarchical AMOVAs (Table 3a) and most of the variation occurred within samples, suggesting that factors other than hosts are likely driving the bulk of the genetic variation. This is further corroborated by the lack of host‐specific lineages within each of the *Peristenus* species (Figure 2). Unlike their herbivore hosts, the two *Peristenus* species exhibit temporal differentiation in Alberta, where both species occur (Figure 2). Both species appear to be attacking all available hosts upon emergence, with *P. mellipes* appearing early in June and attacking the first generation of *Lygus* and *P. howardi* emerging later in August and attacking the second *Lygus* generation. This temporal separation could be the result of selection for niche partitioning to avoid direct competition, as both *Peristenus* species are ecological competitors that occur in the same geographic and host ranges. Alternatively, the presence of this temporal heterogeneity could predate the contact of the two *Peristenus* species; however, this is unlikely as both species collected outside of this contact zone in Alberta are not bound by this strict temporal separation (Zhang et al., 2017). Our findings are consistent with Fernández, Laird, Herle, Goulet, and Cárcamo (2018), who found *P. mellipes* occurs early in the season between late May and late July and *P. howardi* in late June to late August. In addition, emergence times of *P. mellipes* were on average 13 days earlier than *P. howardi* in laboratory trials (Fernández et al., 2018). It is unknown how frequently parasitoids exhibit temporal speciation, as most of previous works on ecological speciation have focused on herbivorous insects (Forbes et al., 2017). However, the development of reproductive isolation as a by‐product of divergent ecological selection should have similar genomewide effects as herbivorous insects, especially if considerable standing genomic variation is already present (Egan et al., 2015; Michel et al., 2010).

**Figure 3:**
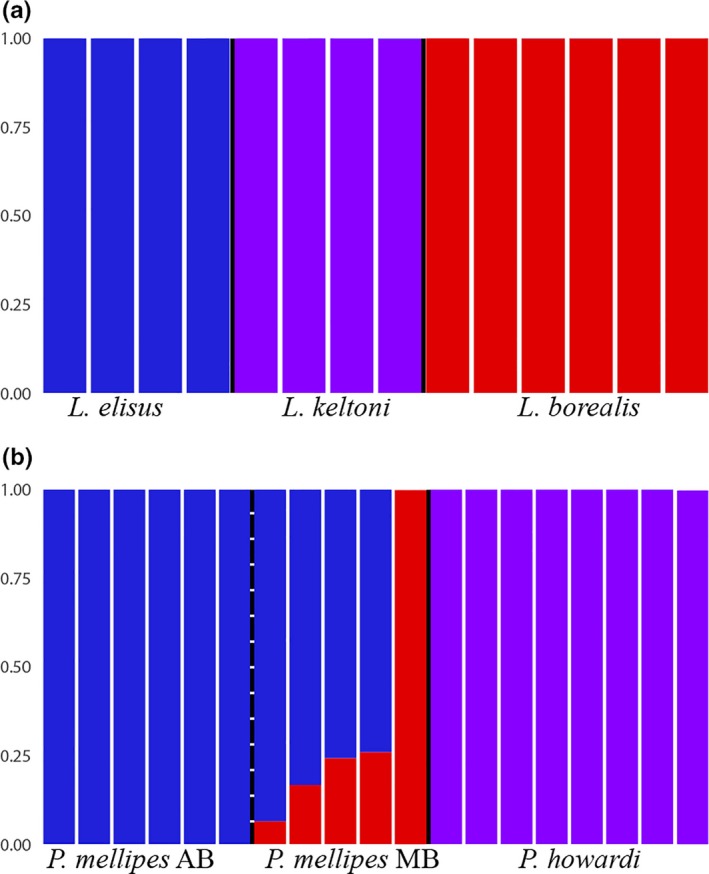
STRUCTURE plots of the full SNP dataset for (a) *Lygus* species collected in Manitoba and Alberta, from three host plants. The most likely number of partitions was *K* = 3 (Δ*K* = 838.45). (b) *Peristenus* species reared from the *Lygus* species collected in (a). The most likely number of partitions was *K* = 3 (Δ*K* = 7,932.06). Solid black lines divide species, and dotted black lines divide populations

Interestingly, both STRUCTURE (Figure 3b) and AMOVA (Table 3b) detected population structure within *P. mellipes* that splits the Manitoba population from Alberta (11.77% variation, *p* = 0.01). However, most of the genetic variation is still within samples of each site (55.78% variation, *p* = 0.01), suggesting that other factors are responsible for the genetic variation observed. Additionally, no host‐associated patterns were observed as Manitoba samples only consisted of wasps reared from *L. borealis* feeding on alfalfa (Table 3). The Manitoba *P. mellipes* has only one generation per year despite the absence of *P. howardi*, which could be the result of their host phenology as Manitoba has a shorter summer than Alberta, thus only allowing for the development of one full generation of *Lygus* (Haye et al., 2013). While *P. mellipes* were only collected from Canadian prairies in this study, previous work (Zhang et al., 2017) and historical records have shown that there are two generations of *Lygus* and *P. mellipes* in warmer regions such as Ontario (Goulet & Mason, 2006). This study is limited in terms of host plant breadth and sampling across the range of both *Peristenus* species; thus, future studies should include additional populations from multiple host plants that cover the entire range of *P. mellipes* to determine the degree of gene flow between the eastern and western populations. The third species within the Nearctic *Peristenus pallipes* complex is *P. dayi*, which emerges earlier than *P. mellipes*, with peak activity late May to early June. *Peristenus dayi* attacks *A. lineolatus* rather than *Lygus* spp. (Goulet & Mason, 2006; Zhang et al., 2017). While *P. dayi* was not the focus of the current study, the effects of partial host and temporal separation between closely related *Peristenus* species and their evolutionary history could be tested using similar methods.

Differences in breeding time can be interpreted as an alternate to spatial differentiation, or as a type of ecological differentiation that warrants further attention, as examples in the literature remain sparse (Taylor & Friesen, 2017). *Peristenus* specialization on different generations of *Lygus* may have led to temporal assortative mating limiting gene flow, equating to allopatric populations separated by temporal rather than physical barriers (Taylor & Friesen, 2017). Without knowing the full distribution range and biogeographic history of these two *Peristenus* species, it is difficult to determine whether temporal separation was the cause of the speciation event or the result of niche partitioning in the form of secondary reinforcement when they came into secondary contact in Alberta. However, in areas such as Manitoba where *Lygus* has one generation per year, we expect that *Peristenus* would show little to no evidence of divergence as there would be little selection pressure on mating/host choice. However, in areas where *Lygus* and *Peristenus* have more than one generation, temporal divergence could facilitate the development of incipient temporal isolation like that shown in this study. In short, studies on whether voltinism facilitates or hampers divergence would yield interesting insights into the broader patterns of herbivore and parasitoid speciation.

## 5 CONCLUSION

Using mitochondrial DNA and genomewide SNPs, our comparative analysis of genetic differentiation between the two sister *Peristenus* species attacking multiple *Lygus* hosts revealed temporal divergence rather than host‐associated differentiation. Temporal isolation likely played a vital role in the speciation process of *Peristenus*, whether it is acting alone or in concert with host preferences or other pre‐ or postzygotic barriers to gene flow. This is one of the first studies to demonstrate the potential of genomic data in resolving the tritrophic evolutionary relationships between plant, herbivore, and parasitoids. This study also demonstrates the importance of systematics to studies of parasitoid speciation, particularly careful delimitation of cryptic species, host rearing to obtain accurate records, and genomic‐scale data for examining any population‐level differences among closely related taxa.

Given the results of our study, the *Lygus*–*Peristenus* system can also be added to the growing body of literature on the importance of temporal separation as a driving force for ecological speciation and its effect on the evolution of the rich diversity of life. Currently, the importance of temporal differences in parasitoid speciation is poorly understood, but temporal isolation likely plays a significant role in the adaptations to host phenology. Many phytophagous insects and their parasitoid systems are well studied because of their agricultural and economical importance; thus, large, collaborative, genomic‐scale studies exploring these taxa could yield valuable insights into the prevalence and impact of temporal isolation in host‐driven ecological speciation.

## CONFLICT OF INTEREST

None declared.

## AUTHOR CONTRIBUTIONS

Y.M.Z. and B.J.S. conceived and designed this project. Y.M.Z., A.I.H.B., and D.C.F. collected specimens. Y.M.Z. and A.I.H.B. generated and analyzed the data. All four authors interpreted the data and wrote the manuscript.

## DATA ACCESSIBILITY

*COI* sequences are available on GenBank Accession Numbers MG944319–MG944389. Raw FastQ files for ddRADseq data can be found on NCBI SRA database Accession Number SRP132595.

The following datasets are available on Dryad (https://doi.org/10.5061/dryad.5vv1dj8):

- VCF files for all raw SNP datasets,
- Input files for MrBayes (Nexus format), RAxML (PHYLIP format), and STRUCTURE (.str format), and
- R scripts for AMOVA and ggtree.
