# Supplementary figures and images for: Habitat or temporal isolation: Unraveling herbivore–parasitoid speciation patterns using double digest RADseq
Source: Ecol Evol. 2018 Sep 12;8(19):9803–16. doi: 10.1002/ece3.4457 (PMC6202701; doi:10.1002/ece3.4457)

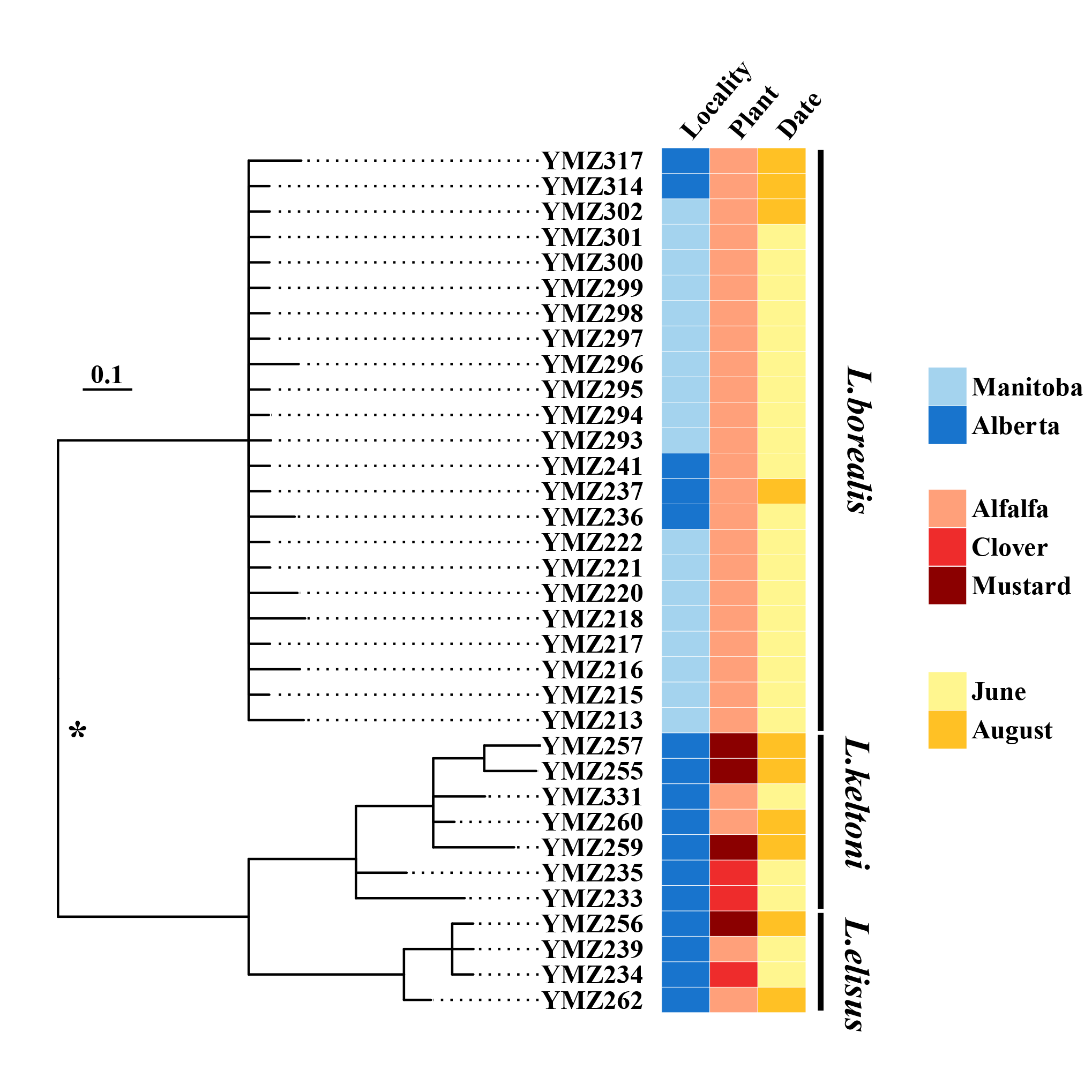

Supplement: Supplementary file 1 [file ECE3-8-9803-s001.tif]

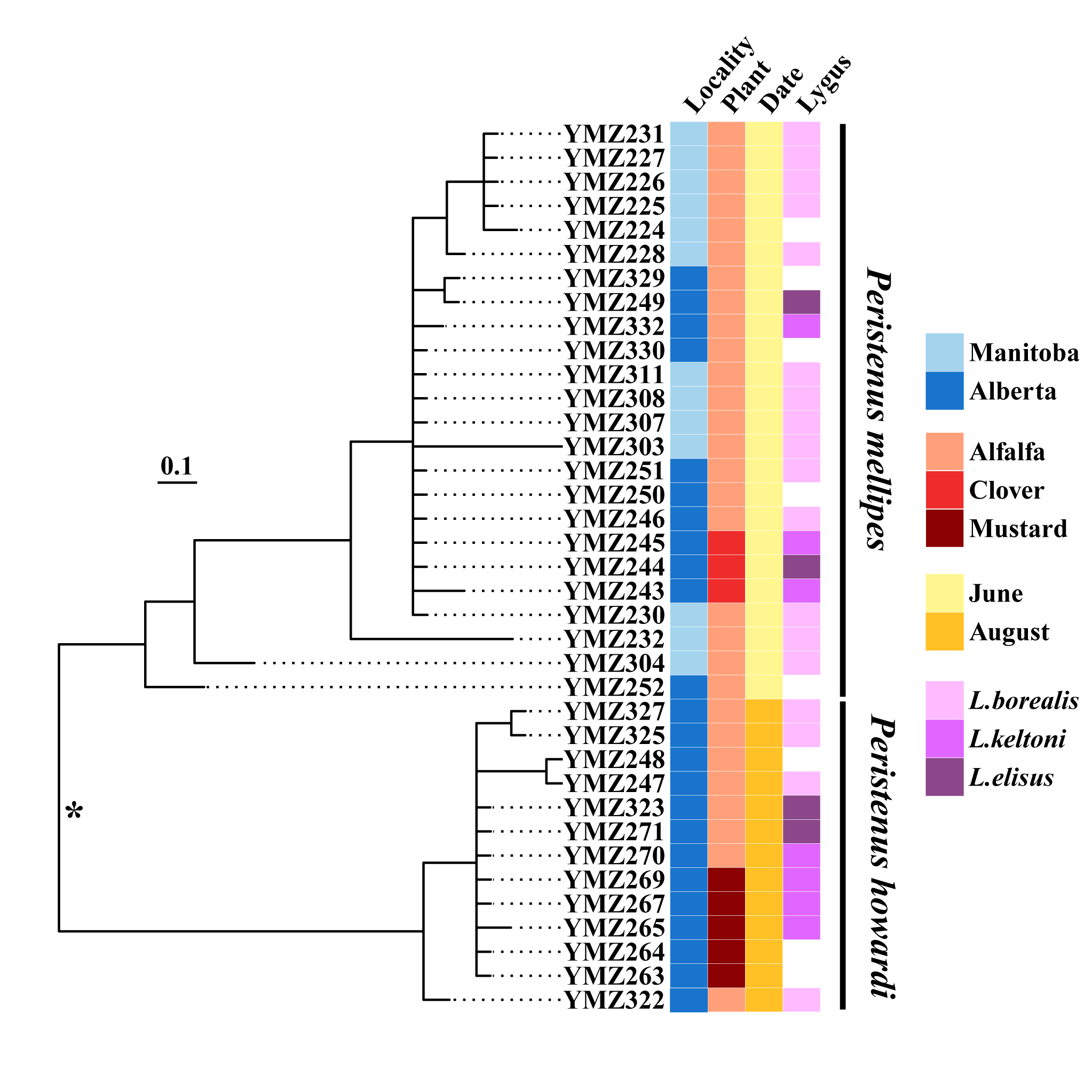

Supplement: Supplementary file 2 [file ECE3-8-9803-s002.tif]
